# On the Formation and Stability of Chitosan/Hyaluronan-Based Complex Coacervates

**DOI:** 10.3390/molecules25051071

**Published:** 2020-02-27

**Authors:** Franco Furlani, Pietro Parisse, Pasquale Sacco

**Affiliations:** 1 Department of Life Sciences, University of Trieste, Via L. Giorgieri 5, I-34127 Trieste, Italy; franco.furlani@phd.units.it; 2 Elettra-Sincrotrone Trieste S.C.p.A., s.s. 14 km 163,500, I-34149 Basovizza, I-34127 Trieste, Italy; pietro.parisse@elettra.eu

**Keywords:** chitosan, hyaluronan, complex coacervation, dissolution/aggregation stability, Small Angle X-ray Scattering (SAXS)

## Abstract

This contribution is aimed at extending our previous findings on the formation and stability of chitosan/hyaluronan-based complex coacervates. Colloids are herewith formed by harnessing electrostatic interactions between the two polyelectrolytes. The presence of tiny amounts of the multivalent anion tripolyphosphate (TPP) in the protocol synthesis serves as an adjuvant “point-like” cross-linker for chitosan. Hydrochloride chitosans at different viscosity average molar mass, $\bar{M_{v}}$, in the range 10,000–400,000 g/mol, and fraction of acetylated units, F_A_, (0.16, 0.46 and 0.63) were selected to fabricate a large library of formulations. Concepts such as coacervate size, surface charge and homogeneity in relation to chitosan variables are herein disclosed. The stability of coacervates in Phosphate Buffered Saline (PBS) was verified by means of scattering techniques, i.e., Dynamic Light Scattering (DLS) and Small-Angle X-ray Scattering (SAXS). The conclusions from this set of experiments are the following: (*i*) a subtle equilibrium between chitosan F_A_ and $\bar{M_{v}}$ does exist in ensuring colloidal stability; (*ii*) once diluted in PBS, osmotic swelling-driven forces trigger the enlargement of the polymeric mesh with an ensuing increase of coacervate size and porosity.

## 1. Introduction

The term “chitosan” recapitulates a class of polysaccharides composed of two building sugars *β*-1→4 linked, i.e., glucosamine (D unit) and *N*-acetyl-glucosamine (A unit), distributed randomly or more block-wise along the polymer chain. Physical-chemical features such as molecular weight and sugar composition influence parameters such as charge density, crystallinity, solubility and susceptibility to enzymatic degradation [1,2]. As an example, chitosan begins to be soluble at neutral pH due to two main reasons: (*i*) the molecular weight of fully deacetylated chitosans is reduced to the point of having few monomers per chain, generally < 10; (*ii*) the fraction of acetylated units, F_A_, of medium-to-high molecular weight chitosans is in the range of 0.4–0.7. To date, chitosans and chitosan derivatives are widely used in different sectors—especially in the biomedical and pharmaceutical fields—to develop systems for Tissue Engineering or Drug Delivery applications [3,4,5,6,7,8,9,10].

All chitosans are, however, soluble in acidic conditions, thanks to the protonation of D-type sugars. In such experimental conditions, chitosans behave as polycations, meaning that they are prone to electrostatically interact with negatively charged multivalent ions, e.g., tripolyphosphate (TPP), or macromolecules, e.g., hyaluronan. This physical phenomenon is defined as “coacervation”, which is of a simple or complex nature, depending on the number of macromolecules involved in the process [11]. Complex coacervation of chitosan or its derivatives, with negatively charged macromolecules, represents a simple method for fabricating an ensemble of colloids amenable to encapsulating and vehiculating payloads, as well as drugs, genes and proteins [12,13,14,15,16,17,18,19,20,21,22,23].

Although chitosan variables—i.e., molecular weight and F_A_—have been deeply investigated to promote complex coacervation in combination with hyaluronans [24], aspects related to dissolution or aggregation colloidal stability in physiological-like media remain hitherto elusive. Chitosan/hyaluronan-based colloids assemble in deionized water and in acidic conditions, typically in the range of pH 4–6, wherein either of the polysaccharides are partially charged. Once moved into media showing physiological ionic strength and pH, the stability of the resulting coacervates might be dramatically limited, due to the screening phenomena of supporting salts and general loss of positive charges on chitosans. In a previous work [24], the authors demonstrated that the addition of metallic Zn(II) preserved the colloidal stability of coacervates. Dissolution or aggregation stability can be eventually improved by varying chitosan molecular weight or the chemical composition, in the case of simple coacervation [25,26]. In our previous contribution, we proved that the stability of chitosan/hyaluronan-based coacervates was ensured using a chitosan with a residual acetylation of 16% and a medium viscosity average molar mass—i.e., $\bar{M_{v}}$ = 220,000 g/mol—in the presence of tiny amounts of the multivalent anion TPP [27].

The goal of this work was to extend our previous findings about the formation and stability of chitosan/hyaluronan-based complex coacervates. Chitosans at different molar masses and chemical compositions, together with a relatively low molar mass hyaluronan, have been selected to fabricate a large library of formulations. The role played by the acetylation degree and chain length of chitosans in forming colloids is herewith disclosed. A combination of light scattering techniques in the visible (Dynamic Light Scattering, DLS) or X-ray domain (Small-Angle X-ray Scattering, SAXS) is finally reported to shed light on the system stability and macromolecular rearrangement upon injection into physiological simulated medium, i.e., Phosphate Buffered Saline (PBS) buffer.

## 2. Materials and Methods

### 2.1. Materials

Hydrochloride chitosans (CHs) with different molar masses (viscosity average molar mass, $\bar{M_{v}}$, in the range of 10,000–400,000 g/mol) and different fractions of acetylated units, F_A_, (determined by ^1^H-NMR) were kindly provided by Novamatrix/FMC Biopolymer (Sandvika, Norway) and by the late Prof. Kjell Morten Vårum (NTNU, Trondheim, Norway). The characteristics of CHs are presented in Table 1. Sodium hyaluronate (HA), ([η] = 270 mL/g; $\bar{M_{v}}$ = 90 000 g/mol, Bioibérica S.A.) was kindly provided by Sigea Srl (Trieste, Italy). Sodium tripolyphosphate pentabasic (TPP ≥ 98%) and Phosphate Buffered Saline (PBS) were from Sigma-Aldrich Co. (St. Louis, MO, USA). All other reagents were from Sigma-Aldrich. All reagents and chemicals were of high purity grade. Deionized Milli-Q water was used in all experiments.

### 2.2. Preparation of Coacervates

The synthesis of coacervates was performed according to a previously published procedure [22]. Briefly, the polymers and TPP were solubilized in deionized water at a concentration equal to 0.6 g/L for CHs, 1.25 g/L for HA and 0.5 g/L for TPP. After complete solubilization, 150 μL of TPP solution were then added dropwise to 3 mL of HA solution under stirring (HA-TPP). The solutions were filtered through 0.22 μm filters (Biosigma, Italy) and stored at room temperature until use. In a 5 mL beaker, 500 μL of the HA-TPP solution were added to 1 mL of the CH solutions under stirring, allowing for the formation of coacervates. The final CH/HA weight ratio was 1:1 [27]. The solutions were kept under stirring for 10 min and left at rest for 20 min, prior to being analyzed.

### 2.3. Physical Characterization of Coacervates

#### 2.3.1. Dynamic Light Scattering (DLS) Analyses

Formulations were studied by means of Dynamic Light Scattering (DLS) on a Zetasizer Nano ZS with 173° detection optics and incident light of 633 nm (Malvern Instruments, UK) to evaluate their size (hydrodynamic diameter), PolyDispersity Index (PDI) and surface charge. Each formulation was analyzed—at least in triplicate—at T = 25 °C, after dilution 1:10 *v/v* in deionized filtered water, using disposable cuvettes. The size was expressed as the Z-average hydrodynamic diameter, obtained by a cumulative analysis of the correlation function, using the viscosity and refractive index of water in the calculations. ζ-potential was determined via the Laser Doppler velocimetry (LDV) technique.

The stability of coacervates was evaluated using PBS—Phosphate Buffered Saline—as the buffer, with a final ionic strength, *I*, of 168 mM and a pH of 7.4 [27,30]. The resulting formulations were analyzed—at least in triplicate—by means of DLS, after dilution 1:10 *v/v* in the buffer. DLS size quality reports, size distribution curves and PDI were considered as parameters to assess the stability of coacervates.

#### 2.3.2. Small Angle X-Ray Scattering (SAXS) Measurements

Small-Angle X-ray Scattering (SAXS) measurements were performed at the Austrian SAXS beamline of the electron storage ring ELETTRA-Sincrotrone Trieste [31] using a photon energy of 8 keV and a beamsize of 0.5 × 2.5 mm. The beamline setup was adjusted to a sample-to-detector distance of 1.00916 m, to provide an accessible *q*-range of 0.086–7.26 nm^−1^. All images were recorded using the Pilatus3 1 M (Dectris, Switzerland), with at least 3 exposures of 10 s each per sample, to check for radiation damage. Reference patterns, to calibrate the *q*-scale, were collected of silver-behenate (d-spacings of 5.838 nm). All measurements were performed using a custom-made flow-through cell with a 1.5 mm X-ray capillary (Hilgenberg, Germany). Radial averaging and image calibration were conducted using the FIT2D software [32]. All presented data were corrected for fluctuations of the primary intensity and transmission; the corresponding background was subtracted from each solution scattering pattern. Coacervates composed by F_A_ = 0.16 and $\bar{M_{v}}$ = 220,000 g/mol chitosan were fabricated and diluted in deionized filtered water or PBS buffer. As a reference, singular polymers (CH and HA), after dilution in the appropriate media (water or PBS), were analyzed as well.

## 3. Results and Discussion

### 3.1. Effect of Chitosan Variables on Coacervates Formation and Physical Properties

Chitosans encompassing molar masses in the range of 10,000–400,000 g/mol were selected to fabricate a large library of coacervates via electrostatic complex coacervation, in combination with HA and TPP. It is worthwhile to initially mention that at whatever the F_A_ considered, all chitosans elicited an onset of turbidity upon the injection of the HA-TPP mixture into the CH solutions, indicating that the formation of visible scatters took place. Limited aggregation, with an ensuing precipitation of the resulting macro-colloids, was observed only in the case of coacervates composed of F_A_ = 0.46 and $\bar{M_{v}}$ = 210,000 g/mol, and F_A_ = 0.63 and $\bar{M_{v}}$ = 310,000 g/mol chitosans (Table 2).

**Figure 1:**
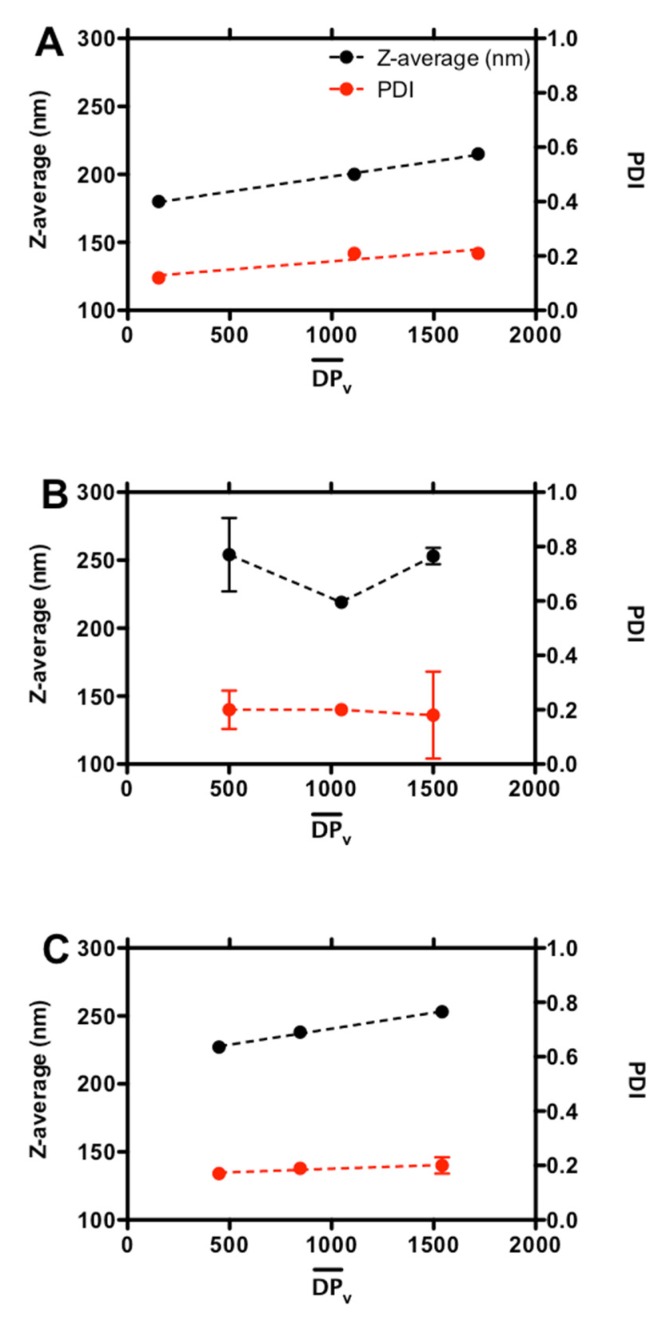
The dependence of the coacervate hydrodynamic diameter (Z-average) and polydispersity index (PDI) on the chitosan degree of polymerization ($\bar{DP_{v}}$), for formulations at different fractions of acetylated units, F_A_: F_A_ = 0.16 (**A**), F_A_ = 0.46 (**B**) and F_A_ = 0.63 (**C**). Dashed lines are drawn to guide the eye.

To study the physical properties of the resulting coacervates (size, polydispersity and surface charge), an in-depth DLS analysis was carried out. In the case of F_A_ = 0.16 and F_A_ = 0.63 chitosans, a linear dependence of the Z-average on the degree of polymerization, $\bar{DP_{v}}$, was detected, meaning that the higher the chitosan molar mass, the higher the hydrodynamic diameter of the related coacervates (Figure 1A,C)—in good agreement with the findings of Delair and co-workers [24]. A slight decrease, followed by a subsequent increment, of coacervate size was observed in the case of F_A_ = 0.46 chitosan (Figure 1B); however, a fair interpretation of the latter results is somewhat speculative due to the presence of large precipitates in the case of $\bar{DP_{v}}$ = 1050, and in general, higher experimental error. Overall, coacervate dimensions were in the range of 180–260 nm whatever the F_A_ considered, suggesting that the acetylation of chitosans had a limited impact on colloid size. A further speculation can be drawn on the basis of the present findings with respect to those of Delair and co-workers. It appears that the presence of tiny amounts of TPP in our synthesis protocol would be beneficial in extending the colloidal domain over that of the aggregation region in a typical $\bar{M_{v}}$ vs. F_A_ plot profile [24].

Whatever the F_A_, all formulations displayed good homogeneity showing PDI ≤ 0.21, in line with similar systems (Table 2) [17,20]. An almost independence of PDI from $\bar{DP_{v}}$ was noticed for coacervates composed of F_A_ = 0.46 and F_A_ = 0.63 chitosans, suggesting that chitosan molar mass did not affect the (overall) homogeneity of coacervates. In the case of colloids composed of F_A_ = 0.16 chitosan, an increment of molar mass from 30,000 to 220,000 g/mol caused a parallel doubling of PDI from 0.12 ± 0.01 to 0.21 ± 0.01; a further increase of molar mass had a very limited effect on the overall coacervate homogeneity.

**Figure 2:**
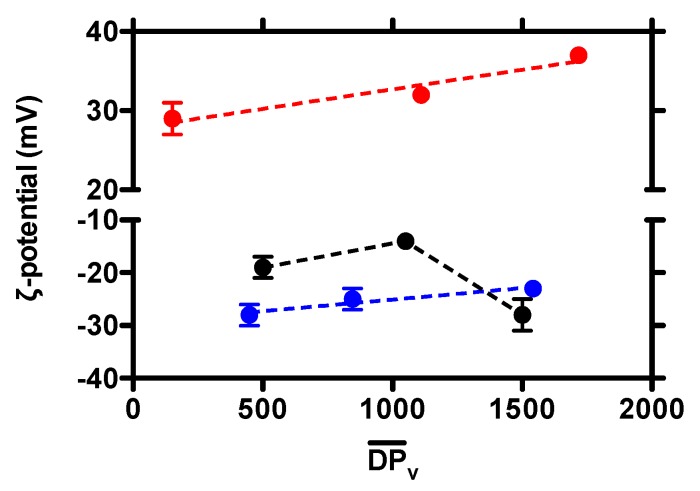
Dependence of coacervate surface charge (ζ-potential) on the chitosan degree of polymerization ($\bar{DP_{v}}$) for formulations at different fractions of acetylated units, F_A_: F_A_ = 0.16 (red dots), F_A_ = 0.46 (black dots) and F_A_ = 0.63 (blue dots). Dashed lines are drawn to guide the eye.

Coacervate surface charge was next investigated by ζ-potential measurements (Figure 2). In the case of colloids composed of F_A_ = 0.16 chitosan, a positive surface charge was recorded, whatever the molar mass considered. Interestingly, a linear dependence of ζ-potential on $\bar{DP_{v}}$ was observed, indicating that the higher the molar mass of chitosan, the higher the whole (positive) surface charge. This finding seems to suggest that an approximately ten-fold increment of chitosan monomers per chain led to a greater abundance of the polycation, i.e., chitosan, at the particle surface, with respect to the core.

A negative surface charge was, at variance, detected for coacervates composed of F_A_ = 0.46 and F_A_ = 0.63 chitosans, in the overall range of molar masses analyzed. This behavior stems from the chemical composition of said chitosans, since the lower the amount of D sugars, the lower the overall positive charge density on the polysaccharide. The main contribution to such a behavior is therefore attributed to the negative charges of HA on the surface of coacervates. Although a linear correlation of ζ-potential with $\bar{DP_{v}}$ was observed for coacervates composed of F_A_ = 0.63 chitosan, more scattered results were recorded in the case of F_A_ = 0.46 chitosan, again likely due to the presence of precipitates.

### 3.2. The Stability of Coacervates as a Function of Chitosan Variables

To evaluate coacervate stability in a physiological medium, coacervates were fabricated in deionized water as usual and diluted in PBS buffer, characterized by a total ionic strength of 168 mM, [phosphate] = 10 mM and pH = 7.4. In the case of coacervates formed by F_A_ = 0.46 and F_A_ = 0.63 chitosans, all coacervates became unstable upon dilution in PBS, whatever the molar mass considered. The instability was determined by means of DLS analyses, which highlighted a remarkable heterogeneity of coacervates (PDI ≥ 0.3) and dimensions spanning from hundreds of nanometers up to microns (Table 3). Overall, all of these formulations displayed partial dissolution and/or aggregation when dispersed in PBS buffer.

**Figure 3:**
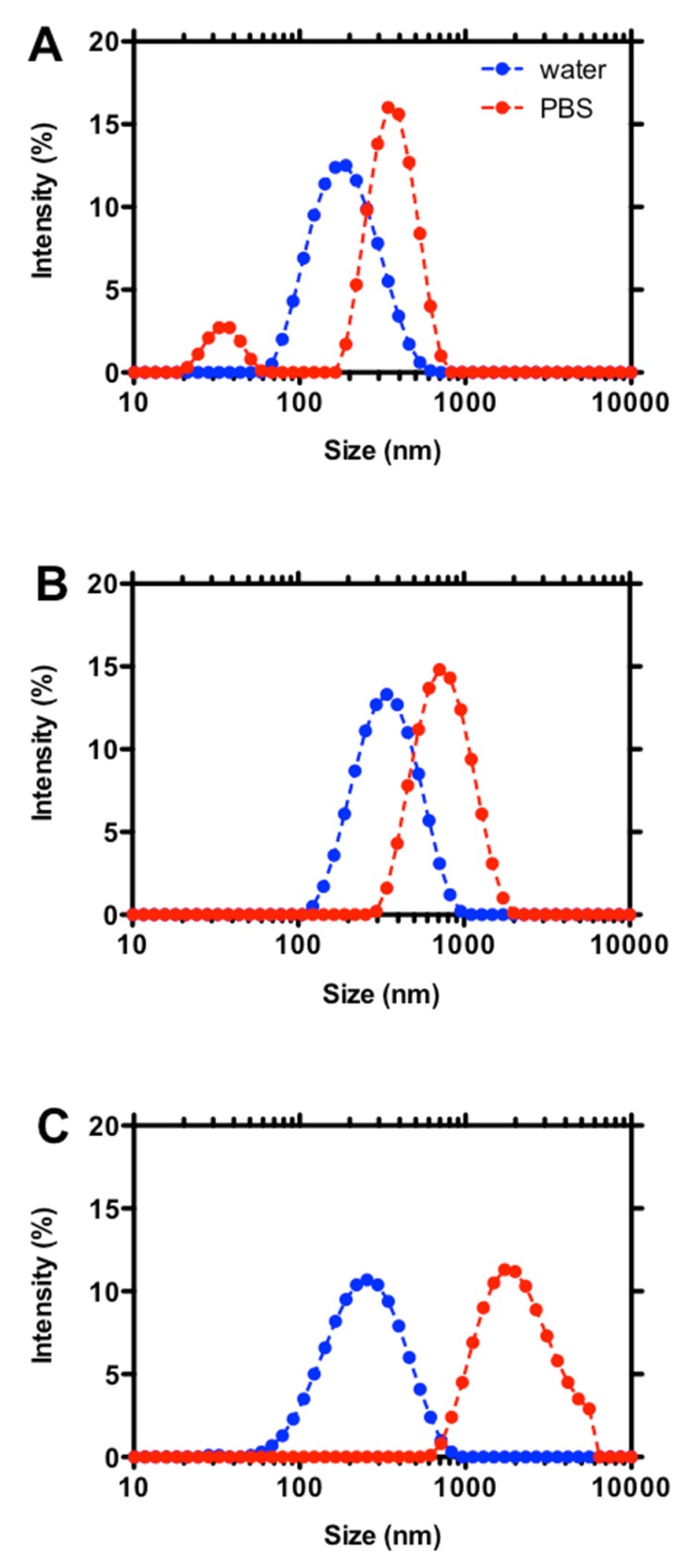
DLS intensity size distribution curves of coacervates composed by F_A_ = 0.16 chitosans at different molar mass (g/mol): $\bar{M_{v}}$= 30,000 g/mol (**A**), 220,000 g/mol (**B**) and 340,000 g/mol (**C**). Coacervates were diluted 1:10 (*v/v*) into two different media prior to measurements, namely deionized water and PBS.

When coacervates composed of F_A_ = 0.16 chitosans are considered, only those formed by medium molar mass chitosan—i.e., $\bar{M_{v}}$ = 220,000 g/mol—remained stable in PBS, in line with our previous findings [27]. Such coacervates displayed a monomodal DLS intensity distribution curve (Figure 3B), albeit shifting toward larger dimensions and greater homogeneity with respect to the same coacervates in unbuffered conditions, i.e., in deionized water.

On the other hand, a reduced degree of polymerization, as well as an increment of chitosan molar mass, marked the instability of the system. Sample-case DLS intensity distribution curves for coacervates composed of $\bar{M_{v}}$ = 30,000 and 340,000 g/mol chitosans are reported in Figure 3A,C, respectively. In the former case, a second less intense peak appeared at smaller dimensions when coacervates were dispersed in PBS, suggesting that partial dissolution took place. For the highest molar mass, the main distribution peak shifted toward very large dimensions. The presence of a shoulder at around 5–6 µm would suggest the formation of a second population of macro-coacervates. In light of these findings, it is clear that a subtle correlation between chitosan physical-chemical characteristics and coacervate stability does exist when a low molar mass hyaluronan is used. Specifically, the joint cooperation of medium acetylation, i.e., 16%, together with an average of around 1100 sugars per chitosan chain, seems necessary to stabilize coacervates in physiological-mimicked conditions without dissolution or severe aggregation.

**Figure 4:**
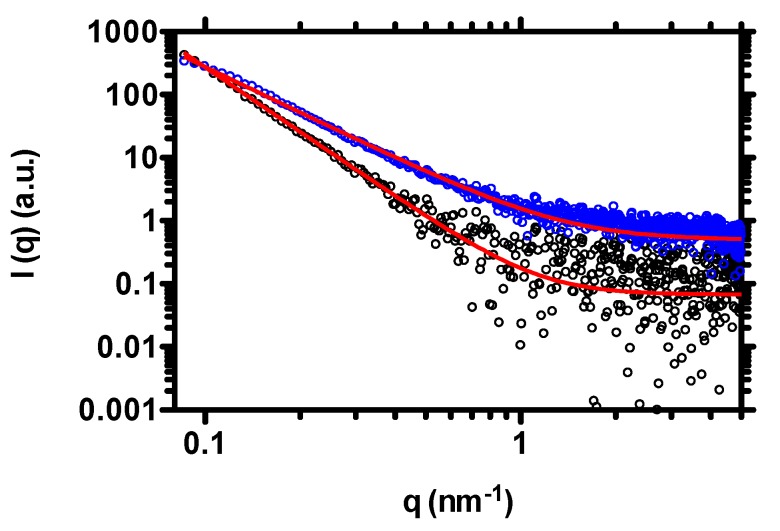
Small-Angle X-ray Scattering (SAXS) pattern profiles of coacervates composed by F_A_ = 0.16 and $\bar{M_{v}}$= 220,000 g/mol chitosan. Coacervates are dispersed in deionized water (black dots) and PBS (blue dots). The final medium composition is the following: 90% *v*/*v* coacervates in deionized water + 10% *v*/*v* deionized water or 10X PBS. The red solid lines represent the best fit of experimental data according to eq. (1).

In our previous contribution, it was demonstrated that pH, total ionic strength and phosphate concentration were at the root of the increase of coacervate size when dispersed in PBS buffer [27]. Among the three parameters, the amount of phosphates was of particular importance, since competition for chitosan binding between the phosphate anions of PBS and TPP was postulated to mark the onset of osmotic swelling, leading to such a dimensional shift. Thus, once diluted in PBS, one might expect that the coacervates rearranged their internal network, from a compact ensemble of polymer chains, towards a more expanded construct. To verify this thesis, the network rearrangement of coacervates was verified by Small-Angle X-ray Scattering (SAXS). Since the dimension of coacervates is outside of the SAXS size window, one can attribute differences in scattering intensity to diverse macromolecular assembly within coacervates. Figure 4 displays the scattering profiles of CH/HA-based coacervates dispersed in both deionized water and PBS.

Both curve profiles do not show any characteristic “polyelectrolyte peak” in the high *q* range, likely due to charge consumption by two polyelectrolytes [33]. Our *q*-range does not allow us to observe the eventual polyelectrolyte peak of CH/HA-based complexes at low *q*, as previously reported by Delair and coworkers [34]. Another consideration can be drawn on the basis of the overall *I(q)* of the two patterns: coacervates in PBS buffer display (in general) similar scattering intensity profiles, with respect to the same colloids in deionized water.

Data analysis was performed by fitting the scattering patterns by using a generalized Porod law Equation (1)

(1) I(q)=k+cpqα

where *I(q)* represents the scattering intensity, *q* is the scattering vector, *k* is a constant, *c_p_* is a pre-factor and α is the Porod exponent, indicating structure compactness. Generally speaking, whereby a Porod exponent of four indicates a compact structure, an exponent between three and four indicates a porous structure [35,36]. The SAXS patterns for single components of coacervates show a Porod exponent of 1.3–2, indicating a rod-like structure (typical of long chains of polymers) [37]. We found that $I\left(q\right)\propto q^{-3.4}$ for coacervates dispersed in deionized water and $I\left(q\right)\propto q^{-2.4}$ for colloids in PBS. The exponents found in our experiments are a clear indication of a different aggregation of the coacervates. The variation of the Porod exponent would suggest that a transition from a more compact polymer mesh into more porous one occurred upon the dilution of the coacervates in PBS buffer. This statement indirectly confirms the limited reduction of scattering intensity at very low *q* values for colloids when dispersed in PBS buffer.

## 4. Conclusions

In this manuscript, we have built upon knowledge about the formation and stability of chitosan/hyaluronan-based complex coacervates. Specifically, we paid special attention to the role played by chitosan variables, such as molar mass and chemical composition, in affecting coacervate dissolution/aggregation stability when dispersed in a physiological medium. This aspect is particularly important for the potential translation of the present system in fields such as nanomedicine. A systematic DLS investigation revealed that a partial acetylation and a medium molar mass of chitosans are essential features to limit the instability of the resulting colloids. On the other hand, SAXS analyses confirmed what was postulated in our previous contribution [27], which was that osmotic swelling—triggered by competition between the phosphates of PBS buffer and TPP—promoted an almost immediate macromolecular rearrangement, with an ensuing widening of the network mesh and an increase of porosity.

## Figures and Tables

**Table 1:** The fraction of acetylated units (F_A_), intrinsic viscosity, [η], viscosity average molar mass, $\bar{M_{v}}$, molar mass of chitosan repetitive unit, $MW_{r.u.}$, and viscosity average degree of polymerization, $\bar{DP_{v}}$, of hydrochloride chitosans used for the synthesis of coacervates. $\bar{M_{v}}$ is calculated using the following Mark–Houwink–Sakurada parameters, i.e., *K* = 8.43 × 10^−3^ mL/g and *a* = 0.92 according to Berth and Dautzenberg [28]. The F_A_ was determined by means of ^1^H-NMR [29], whereas [η] was determined by viscometry.

F_A_	[η]	$\bar{M_{v}}\;(g/\mathrm{mol})$	$MW_{r.u.}\;(g/\mathrm{mol})$	^$\bar{DP_{v}}\;$^
0.63	300	90,000	201	448
	550	170,000		846
	950	310,000		1542
0.46	340	100,000	200	500
	650	210,000		1050
	920	300,000		1500
0.16	110	30,000	198	152
	681	220,000		1111
	1026	340,000		1717

**Table 2:** Characterization of hydrochloride chitosan (CH)/sodium hyaluronate (HA) coacervates after dilution 1:10 (*v*/*v*) in deionized water. Coacervates were fabricated using chitosans at different fractions of acetylated units (F_A_) and viscosity average molar masses ($\bar{M_{v}}$). The hydrodynamic diameter, aggregation at time zero, polydispersity index (PDI) and surface charge, i.e., ζ-potential, (all of them ±SD) of the resulting formulations are reported.

F_A_	$\bar{M_{v}}\;(g/\mathrm{mol})$	Hydrodynamic Diameter (nm)	PDI	ζ-Potential (mV)	Notes
0.16	30,000	180 ± 1	0.12 ± 0.01	29 ± 2	no aggregation
	220,000	200 ± 4	0.21 ± 0.01	32 ± 1	no aggregation
	340,000	215 ± 3	0.21 ± 0.01	37 ± 1	no aggregation
0.46	100,000	254 ± 27	0.20 ± 0.07	−19 ± 2	no aggregation
	210,000	219 ± 4	0.20 ± 0.01	−14 ± 1	limited aggregation
	300,000	253 ± 6	0.18 ± 0.16	−28 ± 3	no aggregation
0.63	90,000	227 ± 1	0.17 ± 0.02	−28 ± 2	no aggregation
	170,000	238 ± 1	0.19 ± 0.02	−25 ± 3	no aggregation
	310,000	253 ± 3	0.20 ± 0.03	−23 ± 1	limited aggregation

**Table 3:** Characterization of CH/HA coacervates after dilution 1:10 (*v*/*v*) in Phosphate Buffered Saline (PBS) buffer. Coacervates were fabricated using chitosans at different fractions of acetylated units (F_A_). The hydrodynamic diameter, stability at time zero and polydispersity index (PDI) (all of them ± SD) of the resulting formulations are reported.

F_A_	$\bar{M_{v}}\;(g/\mathrm{mol})$	Hydrodynamic Diameter (nm)	PDI	Notes
0.16	30,000	[270 ± 42] (*)	[0.40 ± 0.03] (*)	unstable
	220,000	773 ± 21	0.08 ± 0.07	stable
	340,000	[1307 ± 461] (*)	[0.35 ± 0.36] (*)	unstable
0.46	100,000	[194 ± 38] (*)	[0.49 ± 0.23] (*)	unstable
	210,000	[982 ± 743] (*)	[0.76 ± 0.28] (*)	unstable
	300,000	[213 ± 49] (*)	[0.32 ± 0.06] (*)	unstable
0.63	90,000	[1060 ± 823] (*)	[0.82 ± 0.20] (*)	unstable
	170,000	[1037 ± 21] (*)	[0.38 ± 0.04] (*)	unstable
	310,000	[103 ± 24] (*)	[0.66 ± 0.02] (*)	unstable

(*) stands for formulations where significant signal errors in the size quality report were detected by Dynamic Light Scattering (DLS) analyses.
